# Marginal Fit, Internal Adaptation, and Microleakage of Preformed Metal Crowns: Impact of Three Preparation Techniques and Three Ion-Releasing Cements

**DOI:** 10.3390/children13091187

**Published:** 2026-09-03

**Authors:** Sanaa N. Al-Haj Ali, Haneen Alsamaani, Shatha Aldhilan, Ruba Alodaib

**Affiliations:** 1 Department of Orthodontics and Pediatric Dentistry, College of Dentistry, Qassim University, Al-Mulaydah 52571, Saudi Arabia; 2 College of Dentistry, Qassim University, Al-Mulaydah 52571, Saudi Arabia; 3 Department of Pediatric Dentistry, Second Cluster RC2, Ministry of Health, Riyadh 13316, Saudi Arabia

**Keywords:** bioactive cement, bioceramic cement, crowns, Hall technique, marginal fit, microleakage, modified Hall technique, primary teeth

## Abstract

**Highlights:**

**What are the main findings?**

• Preparation technique, not cement, governed the performance of preformed metal crowns (PMCs): the Hall technique showed significantly greater microleakage and a larger angular internal gap than conventional preparation, with the modified Hall technique being intermediate; marginal gap was similar across techniques.
• The bioactive (ACTIVA^™^ BioACTIVE-CEMENT) and bioceramic (Calibra^®^ Bio) cements performed comparably to resin-modified glass ionomer cement.

**What are the implications of the main findings?**

• No differences in seal or fit were detected among the three ion-releasing cements.
• Tooth preparation improves the crown–tooth seal and angular fit under PMCs compared with no preparation, supporting clinical evaluation of a modified Hall technique with a defined occluso-axial bevel.

**Abstract:**

Objectives: To compare, in vitro, the marginal and internal fit and marginal microleakage of preformed metal crowns (PMCs) placed with the conventional, Hall, and modified Hall techniques and cemented with three ion-releasing, resin- or glass-ionomer-matrix luting materials: a bioactive resin-matrix cement (ACTIVA^™^ BioACTIVE-CEMENT), a bioceramic calcium aluminate cement (Calibra^®^ Bio), and a resin-modified glass ionomer cement (RMGIC; Riva Luting Plus). Methods: Forty-five extracted human primary molars were randomly allocated to three preparation groups (n = 15), each subdivided by cement (n = 5). After thermocycling (500 cycles, 5–55 °C, dwell time: 15 s, transfer time: 10 s), specimens were immersed in 1% methylene blue for 24 h, sectioned buccolingually, and examined by image analysis (ImageJ). Microleakage was quantified as the percentage of the crown–tooth interface showing dye penetration; marginal and internal fit were measured at thirteen points across marginal, axial, angular, and occlusal regions. Results: Marginal gap did not differ significantly among techniques or cements (*p* > 0.05). A significant region × technique interaction (*p* = 0.025) localised the technique effect to the angular region, where the Hall technique produced a larger gap than conventional preparation (*p* = 0.004). Microleakage was significantly greater for the Hall than the conventional technique (*p* = 0.045), with the modified Hall being intermediate. Cement had no significant effect, and fit and microleakage were uncorrelated. Conclusions: Preparation technique, rather than cement, was the principal determinant of seal and angular fit; ACTIVA^™^ BioACTIVE-CEMENT and Calibra^®^ Bio performed comparably to RMGIC under the conditions tested. Clinical studies of a modified Hall technique incorporating a defined occluso-axial bevel are warranted to confirm these findings.

## 1. Introduction

Full-coverage restorations are indicated in paediatric dentistry for multisurface caries, developmental defects, pulp-treated teeth, space-maintainer abutments, and high-caries-risk patients [1]. Preformed metal crowns (PMCs) are durable, efficient, and cost-effective, and allow trimming and crimping for optimal fit [2]. They outperform amalgam and resin-based restorations for multisurface lesions in young children and are the American Academy of Pediatric Dentistry (AAPD)-recommended restoration for high-caries-risk children [3].

Several techniques exist for PMC placement. The conventional technique requires local anaesthesia and reduction of the occlusal and proximal surfaces, with buccolingual reduction usually limited to a bevel at the occlusal third [4,5]. The Hall technique instead seats and cements the crown over a carious primary molar without local anaesthesia, caries removal, or tooth preparation, producing a transient open bite that usually resolves within a month. It offers a minimally invasive intervention for pre-cooperative children, anxious patients, those with special healthcare needs or limited access to care, and those for whom a definitive restoration is not possible (e.g., long general-anaesthesia waiting lists or lack of insurance) [6].

Modified Hall techniques add minimal proximal preparation (proximal slicing), with or without cusp reduction, to ease seating where tight proximal contacts would hinder fit [1,7,8]. In a recent randomised trial, Turker and Cihan [9] proposed partial caries excavation before cementation, reporting two-year success comparable to the original Hall technique; notably, Hall-treated teeth with insufficient crown fit showed significantly more major failures than those with adequate fit.

Marginal and internal adaptation help determine a PMC’s clinical durability. A marginal discrepancy exposes the luting cement to the oral environment and accelerates its dissolution, and the clinically acceptable gap (approximately 100–150 μm) is hard to attain in PMCs, whose form can be adjusted only slightly by contouring and crimping [10]. Because adaptation depends largely on preparation and seating, technique strongly influences both fit and the marginal seal governing microleakage [4,10], whose reduction lowers the risk of recurrent caries, pulpal pathology, and failure from coronal leakage [4].

The luting cement is the other determinant of the seal. Resin-modified glass ionomer cement (RMGIC) is well established for PMCs [1], valued for its adhesion and fluoride release. More recently, bioactive resin-based (e.g., ACTIVA^™^ BioACTIVE-CEMENT) and bioceramic calcium aluminate (e.g., Calibra^®^ Bio) cements have emerged, offering biocompatibility, ease of handling, and ion-release chemistry proposed to promote hydroxyapatite formation and enhance the marginal seal. Both are bioactive—releasing ions and capable of forming hydroxyapatite according to their manufacturers—differing in matrix chemistry: an ionic resin matrix versus a calcium aluminate ceramic matrix. The cements also differ in polymerisation mode: ACTIVA^™^ BioACTIVE-CEMENT is dual-cure, whereas Calibra^®^ Bio and the RMGIC (Riva Luting Plus) set by self-cure alone. They are not interchangeable, however: resin-matrix bioactive cements showed lower microleakage than calcium aluminate cements in permanent-tooth zirconia crowns [11], yet whether they match RMGIC under PMCs is unknown. Their sealing chemistry may be most relevant to the Hall and modified Hall techniques, which seat the crown over carious teeth of varying lesion depths. Fluoride release from ion-releasing materials has been associated with reduced enamel demineralisation adjacent to bonded metallic attachments [12], and unlike conventional glass ionomer, RMGIC, or resin cements, which do not release calcium and phosphate and therefore cannot precipitate hydroxyapatite, the bioactive and bioceramic cements release calcium, phosphate, and fluoride ions, giving them the potential not only to seal the lesion but also to support remineralisation of the underlying carious dentine [13].

Hall-placed crowns show greater marginal gaps and microleakage than conventionally placed ones, with fit governed by technique rather than luting agent [14], a pattern echoed by a later study in which conventional placement again leaked least [15]. Neither the modified Hall technique nor bioactive and bioceramic cements have been evaluated for these outcomes. These questions bear directly on chairside decisions. When a paediatric dentist faces a young or pre-cooperative child at high caries risk in whom local anaesthesia and conventional preparation are impractical, the decision often shifts to the minimally invasive Hall crown; however, where tight proximal contacts prevent seating and would require prior orthodontic separator placement, and the family cannot return for a second visit to cement the PMC, a modified Hall approach with proximal slicing may be adopted. This matters because fit is not merely a technical outcome: Hall-placed crowns with insufficient fit have shown substantially higher rates of major failure [9], and a compromised marginal seal permits reactivation of the sealed lesion. Similarly, because the Hall and modified Hall techniques seat crowns over retained carious dentine, cements releasing calcium and phosphate might be expected to offer an advantage in precisely this application, yet no evidence supports selecting them over RMGIC under PMCs. This study therefore compared the marginal and internal fit and microleakage of PMCs placed with conventional, Hall, and modified Hall techniques and cemented with three luting cements (bioactive, bioceramic, and RMGIC). The null hypotheses were that fit and microleakage would not differ among techniques or cements and that no technique–cement interaction would exist.

## 2. Materials and Methods

### 2.1. Sample Size Calculation

The sample size was estimated using G*Power (version 3.1.9.7; Heinrich-Heine-Universität Düsseldorf, Germany) for a two-way ANOVA with preparation technique and luting cement as fixed factors, with marginal microleakage as the primary outcome. Based on a comparable in vitro study of luting-cement microleakage under PMCs in primary molars, which reported large between-cement differences in dye penetration [16], a large effect size (f = 0.60) was assumed, with α = 0.05 and power (1 − β) = 0.80. The analysis indicated a minimum total sample size of 31; this was increased to 45 to ensure balanced allocation—15 per preparation-technique group, subdivided into 5 per luting cement across the nine technique–cement subgroups. This total is consistent with Gundewar et al. [16], who used 45 teeth (15 per cement group).

### 2.2. Sample Collection and Preparation

This in vitro study was approved by the Committee of Research Ethics at Qassim University (reference no. 24-08-05, approval date: 8 October 2024). Forty-five extracted human primary molars were included (sound or carious, resorption below two-thirds). Teeth were stored in distilled water at 23 ± 1 °C for up to one month, with the storage water renewed weekly. Teeth which had buccal or lingual caries were excluded; remaining carious teeth were cleaned, leaving intact, caries-free buccal and lingual surfaces. In teeth with pulp exposure, pulp remnants were removed and the pulp chamber was filled with RMGIC (RIVA Light cure restorative material, SDI, Bayswater, Australia) and the cavity was restored to its original form with composite resin (Filtek^™^ Z 350 XT enamel A1 shade, 3M ESPE, St. Paul, MN, USA) after a 20 s etch-and-rinse technique (Any-Etch^®^ Gel, Medental International Inc., Miami, FL, USA) and bonding (Stae total etch adhesive, SDI, Australia). Polymerisation steps were 10 s for the adhesive and 20 s for each layer of resin composite using a light-emitting diode (LED) (Acteon Mini LED^™^ Super-Charged, Acteon Group, La Ciotat, Bouches-du-Rhône, France; radiant intensity: 2000 mW/cm^2^; wavelength range: 420–480 nm).

Before treatment, the root portion up to 2 mm below the cemento-enamel junction (CEJ) was coated with 2 layers of nail varnish. Then, each tooth was embedded in clear cold-cure acrylic resin (Eco Cryl Cold, Protechno, Spain) in an upright orientation up to 2 mm below the CEJ, using a silicone cube mould (2 × 2 × 1 cm).

### 2.3. Crown Preparation and Cementation

The teeth were randomly allocated to three main groups (n = 15) according to preparation technique via a computer-generated randomisation list, with nine subgroups (n = 5) defined by the luting cement used. All preparations were performed by a single calibrated operator. To eliminate variation in cutting efficiency, each diamond bur was replaced with a new one after every ten preparations.

First group (conventional preparation): The mesiodistal dimension of each tooth was measured with a periodontal probe to determine the appropriately sized PMC (3M^™^ ESPE^™^ Stainless Steel Crowns, 3M ESPE). Mesial and distal surfaces were prepared using a needle diamond bur (NTI-Kahla GmbH, Kahla, Germany) with 1 mm reduction from each surface with a feather-edge finish line without ledges, while occlusal reduction of 1–1.5 mm following the cuspal anatomy was done using a football diamond bur (NTI-Kahla GmbH, Germany). Buccal and lingual surfaces received a minimal bevel at the occlusal third and all line angles were rounded. The selected pre-contoured and pre-trimmed PMC was fitted and crimped with pliers (no. 800112 and 800417, 3M ESPE^™^).

Second group (modified Hall technique): Minimal preparation was carried out, limited to reduction of mesial and distal surfaces by a total of 1 mm using a needle diamond bur and minor occlusal cusp tip reduction using a football diamond bur (approximately 0.5 mm) in accordance with the protocol described by Ludwig et al. [1] and Midani et al. [7]. A pre-contoured, pre-trimmed PMC was seated as for the first group.

Third group (Hall technique): No tooth preparation was performed; a pre-contoured, pre-trimmed PMC was seated as for the first group.

Within each preparation group, specimens were further subdivided (n = 5) according to the luting cement employed (Table 1):

• Subgroup A: Bioactive ionic resin-matrix cement (dual-cure)—ACTIVA^™^ BioACTIVE-CEMENT (Pulpdent Corp., Watertown, MA, USA);
• Subgroup B: Bioactive calcium aluminate bioceramic cement (self-adhesive)—Calibra^®^ Bio (Dentsply Sirona, Charlotte, NC, USA);
• Subgroup C: Resin-modified glass ionomer cement—Riva Luting Plus (SDI Limited, Bayswater, Australia).

Each cement was dispensed and manipulated in accordance with the respective manufacturer’s instructions for use.

Subgroup A (ACTIVA^™^ BioACTIVE-CEMENT): A mixing tip was placed on the automix double-barrel syringe, and 1–2 mm of cement was first expressed onto a mixing pad and discarded to ensure a homogeneous base–catalyst mix. The cement was then dispensed directly into the internal surface of the crown, which was immediately seated on the prepared tooth.

Subgroup B (Calibra^®^ Bio): The internal (intaglio) surface of each crown was air-abraded with 50 µm aluminium oxide for 60 s using an air-abrasion handpiece, then cleaned and dried. The capsule was activated by firmly depressing the plunger against a hard surface and immediately triturated for 8 s at high speed (≈4500 rpm). The mixed cement was extruded into the crown and seated within the 2-min working time.

Subgroup C (Riva Luting Plus): The capsule was activated and triturated for 10 s in an amalgamator. Cement was extruded into the crown and seated within 30 s of mix completion.

To standardise seating, each crown was fully seated by finger pressure, after which a static axial load of 40 g was applied for 10 min to maintain the crown in position undisturbed during setting. After initial setting (tack-cure for ACTIVA^™^ at 1–2 s; gel stage at approximately 2 min for Calibra^®^ Bio and Riva Luting Plus), excess cement was removed from the margins with an explorer. For ACTIVA^™^ BioACTIVE-CEMENT, final polymerisation was completed by light-curing each surface at the margin area for 20 s; Calibra^®^ Bio and Riva Luting Plus were allowed to self-cure undisturbed for 6–8 min and 4.5 min, respectively, with isolation maintained until full set.

After cementation, all specimens were stored in distilled water for 24 h. Specimens were then subjected to thermocycling for 500 cycles between 5 ± 2 °C and 55 ± 2 °C, with a dwell time of 15 s and a transfer time of 10 s.

### 2.4. Microleakage Assessment

Following thermocycling, the specimens were immersed in 1% methylene blue dye solution (Biopharm, Inc., Hatfield, AR, USA; manufacturer-specified pH 3.0–4.5) at 37 °C for 24 h. After immersion, specimens were thoroughly rinsed with distilled water, then air-dried, and the crowns were embedded in clear cold-cure acrylic resin.

**Figure 1:**
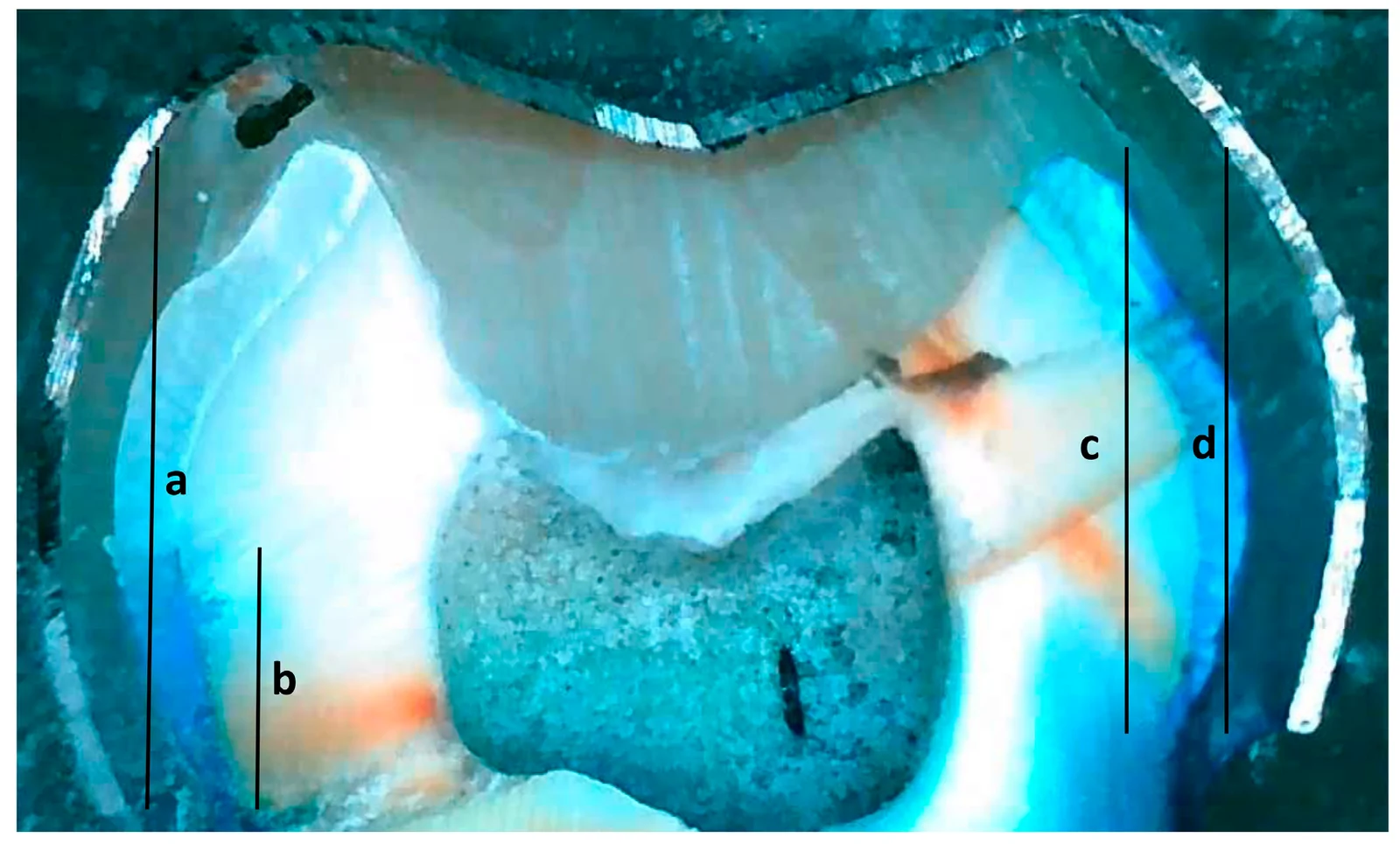
Representative buccolingual section of a preformed metal crown–tooth specimen viewed under stereomicroscopy (×20) after immersion in 1% methylene blue. Vertical bars mark (a) total interface length at the buccal surface, (b) extent of dye penetration at the buccal surface, (c) total interface length at the lingual surface, and (d) extent of dye penetration at the lingual surface. Microleakage was quantified as the extent of dye penetration along each crown–tooth interface expressed as a percentage of the total interface length, measured separately at the buccal and lingual surfaces using image-analysis software (ImageJ).

Each tooth was sectioned longitudinally in a buccolingual direction into two halves using a low-speed diamond disc (NTI-Kahla GmbH, Germany) under copious water irrigation, exposing the crown–tooth interface on both the buccal and lingual aspects of each half. The sectioned surfaces were examined under a stereomicroscope (Hamilton International s.r.l., Lazio, Italy) at ×20 magnification, and digital photographs were captured for each section. The extent of dye penetration along each interface was measured as the percentage of the total interface length showing dye infiltration (Figure 1). Sections demonstrating dye penetration extending across the entire buccal or lingual interface or reaching the occlusal aspect were assigned a microleakage value of 100% [4]. All measurements were performed independently by a single blinded examiner using image-analysis software (ImageJ 1.54t, NIH, Bethesda, MD, USA). For each tooth, the readings from the two halves were averaged to yield a single buccal and a single lingual microleakage value, and the mean of these two values was used as the per-specimen score for statistical analysis.

### 2.5. Marginal and Internal Fit Assessment

**Figure 2:**
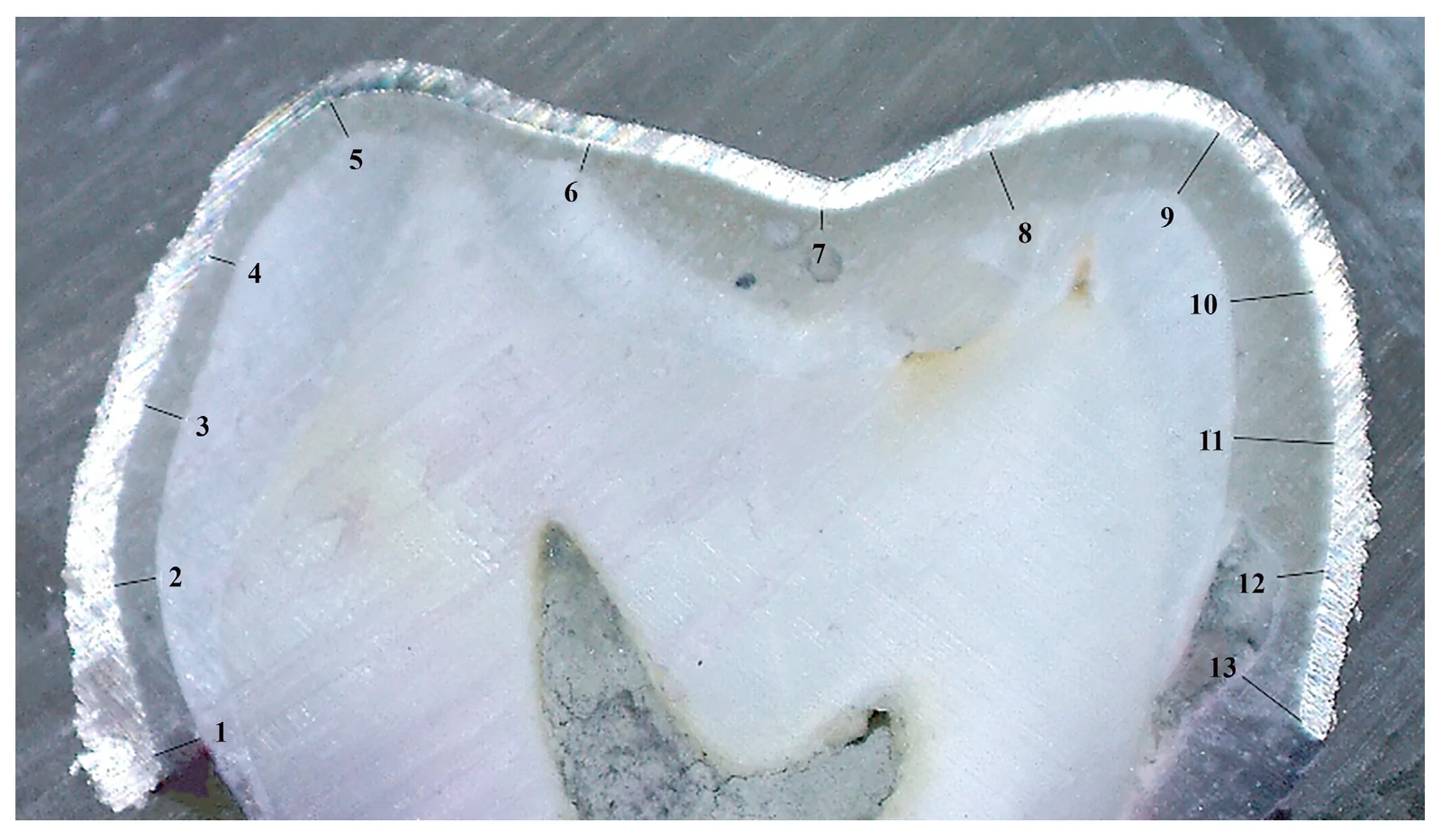
Representative buccolingual section showing the thirteen predefined points at which marginal and internal fit (the perpendicular crown–tooth distance) were measured: marginal gap, points 1 and 13; axial gap, points 2–4 and 10–12; angular gap, points 5 and 9; occlusal gap, points 6–8.

Marginal and internal fit were assessed on the same buccolingual sections used for microleakage evaluation using ImageJ by a single blinded examiner, following a modification of the method described by Al-Haj Ali [10] with the measurement points classified into preparation-based regions in accordance with Wu et al. [18]. For each section, the perpendicular distance between the internal (intaglio) surface of the crown and the corresponding tooth surface was measured at thirteen predefined points distributed across four regions: two marginal (the buccal and lingual crown margins), six axial (three along each of the buccal and lingual axial walls), two angular (the buccal and lingual line-angle transitions between the axial and occlusal surfaces), and three occlusal (across the occlusal surface) (Figure 2). The marginal gap was defined as the perpendicular distance between the crown margin and the tooth at the crown edge, whereas the internal gap was defined as the perpendicular distance between the internal crown surface and the underlying tooth structure, recorded separately for the axial, angular, and occlusal regions [18].

For each specimen, measurements were averaged within each region across the two sectioned halves to yield a single per-tooth value for the marginal, axial, angular, and occlusal regions. Intra-examiner reliability for all outcomes was assessed by re-measuring a randomly selected 20% of sections (n = 9) after an interval of two weeks.

### 2.6. Statistical Analysis

Data were analysed using IBM SPSS Statistics (version 26; IBM Corp., Armonk, NY, USA), with the level of significance set at α = 0.05. Normality was assessed by applying the Shapiro–Wilk test to the standardised residuals of each model, and homogeneity of variance with Levene’s test. Marginal gap was analysed using a two-way ANOVA, with preparation technique and luting cement as fixed factors, followed by Bonferroni-adjusted pairwise comparisons.

Internal gap was analysed using a mixed-model repeated-measures ANOVA in which internal region (axial, angular, occlusal) served as the within-subject factor and preparation technique and luting cement as the between-subject factors. Sphericity was verified with Mauchly’s test, and the Greenhouse–Geisser correction was applied where required; significant interactions were explored through simple-effects analysis with Bonferroni-adjusted pairwise comparisons.

Microleakage was analysed using a two-way ANOVA. As the technique groups exhibited unequal variances, the effect of preparation technique was additionally evaluated using Welch’s ANOVA with Games–Howell post hoc comparisons. The relationship between fit and microleakage was examined using Spearman’s rank-order correlation. Intra-examiner reliability was evaluated using the intraclass correlation coefficient (two-way mixed model, absolute agreement, single measures).

## 3. Results

Prior to analysis, statistical assumptions were verified. Standardised residuals satisfied normality for all outcomes (Shapiro–Wilk, all *p* ≥ 0.060), and no data transformation was required. The internal-gap data satisfied sphericity (Mauchly W = 0.87, *p* = 0.094) and equality of covariance matrices (Box’s *M*, *p* = 0.108). Intra-examiner reliability on a subset of 20% of sections showed intraclass correlation coefficient values of 0.818 for marginal gap, 0.936 for axial gap, 0.913 for occlusal gap, 0.806 for angular gap, and 0.998 for microleakage.

Marginal gap: Two-way ANOVA revealed no significant effect of preparation technique (F_2,36_ = 0.81, *p* = 0.454, η*p*_2_ = 0.043), luting cement (F_2,36_ = 0.41, *p* = 0.664, η*p*_2_ =0.023), or their interaction (F_4,36_ = 1.95, *p* = 0.123, η*p*_2_ = 0.178) on marginal gap (Table 2).

**Figure 3:**
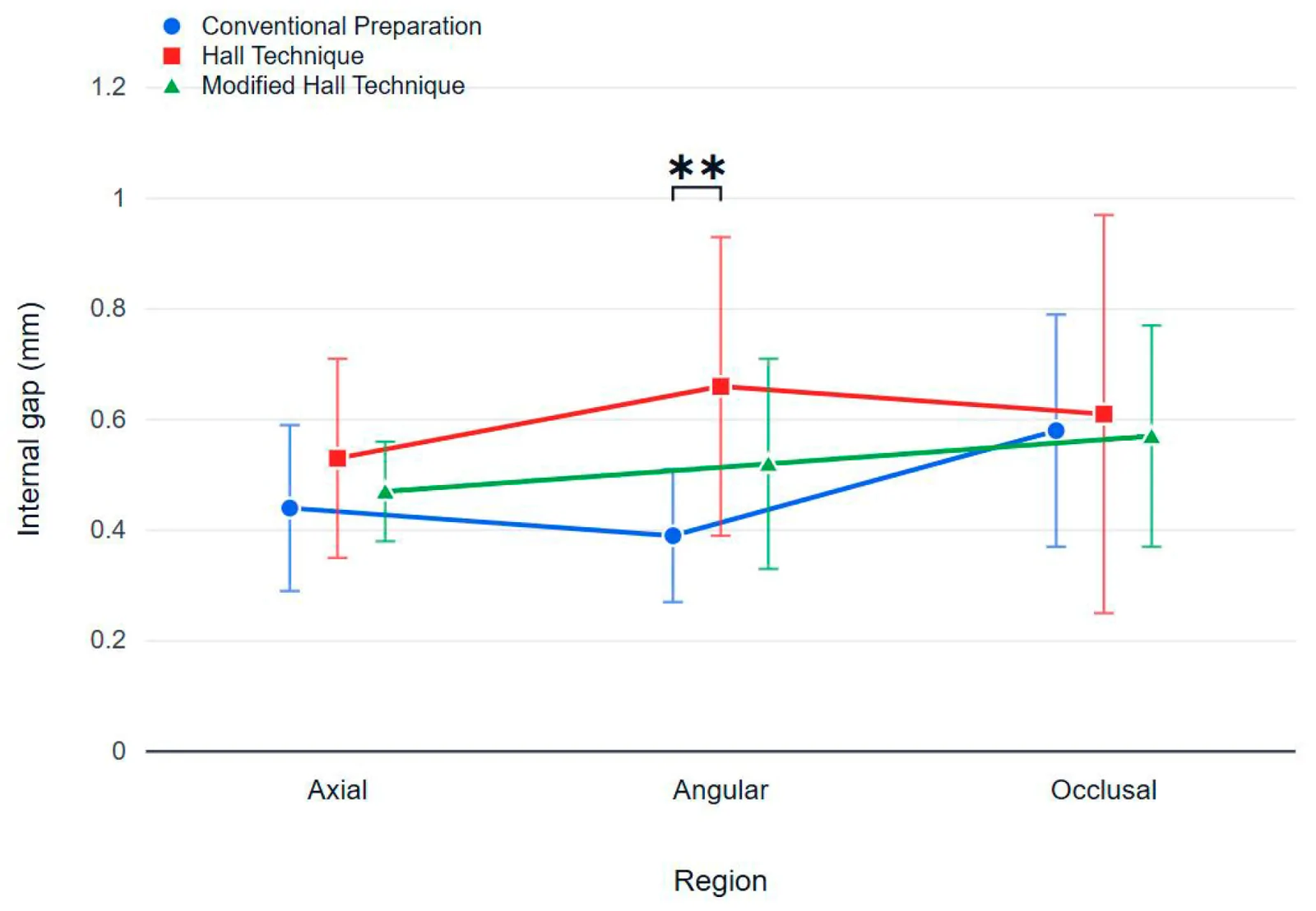
Internal gap (mm; mean ± SD) by tooth region (axial, angular, occlusal) for the conventional, Hall, and modified Hall techniques. ** *p* < 0.05.

Internal gap: Repeated-measures ANOVA showed a significant main effect of region (F_2,72_ = 6.30, *p* = 0.003, η*p*_2_ = 0.149), with the occlusal region exhibiting the largest discrepancy and the axial region the smallest (Table 2). The region × preparation-technique interaction was significant (F_4,72_ = 2.96, *p* = 0.025, η*p*_2_ = 0.141). Simple-effects analysis localised this effect to the angular region (F_2,36_ = 6.05, *p* = 0.005, η*p*_2_ = 0.252), in which the Hall technique produced a significantly larger gap than conventional preparation (*p* = 0.004); no significant technique effect was detected in the axial (*p* = 0.256) or occlusal (*p* = 0.930) regions (Figure 3). Neither the main effect of luting cement (F_2,36_ = 0.49, *p* = 0.617) nor the region × cement interaction (F_4,72_ = 1.19, *p* = 0.325) was significant, and the overall (between-subject) effect of preparation technique did not reach significance (F_2,36_ = 1.80, *p* = 0.180).

**Figure 4:**
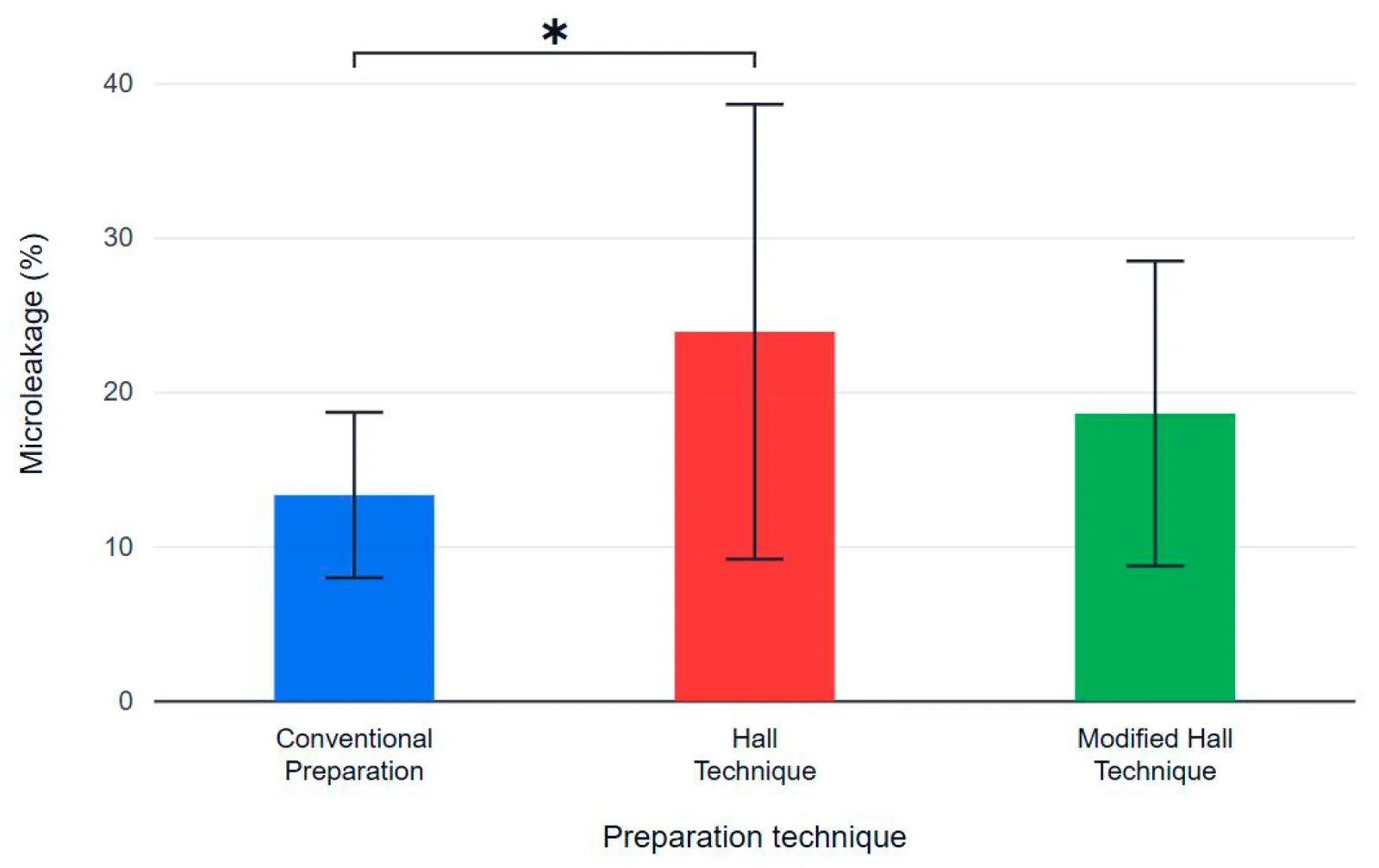
Marginal microleakage (%; mean ± SD) by preparation technique. * *p* < 0.05.

Microleakage: Two-way ANOVA showed no significant effect of luting cement (F_2,36_ = 0.40, *p* = 0.672) or the technique × cement interaction (F_4,36_ = 0.38, *p* = 0.824) on microleakage. As the technique groups exhibited unequal variances (Levene, *p* = 0.006), the effect of preparation technique was evaluated using Welch’s ANOVA, which was significant (F_2, 24.21_ = 4.29, *p* = 0.025). Games–Howell comparisons confirmed significantly greater microleakage with the Hall technique than with conventional preparation (*p* = 0.045); the modified technique was intermediate and did not differ significantly from either (Table 2; Figure 4).

Correlation between fit and microleakage: Spearman correlation showed no significant association between microleakage and marginal gap (*ρ* = −0.11, *p* = 0.473) or any internal region (axial *ρ* = −0.04, *p* = 0.813; angular *ρ* = 0.29, *p* = 0.052; occlusal *ρ* = 0.09, *p* = 0.569).

## 4. Discussion

This study compared the marginal and internal fit and the marginal microleakage of PMCs placed with the conventional, Hall, and modified Hall techniques, and cemented with three luting cements. Microleakage was assessed using the dye penetration technique, which is widely used in the dental literature for this purpose [4,19,20,21]. A notable variation from the clinical protocol, consistent with Erdemci et al. [14], was the caries removal and restoration of specimens in the Hall and modified Hall groups. This was done to standardise the crown–tooth interface across groups and eliminate substrate heterogeneity as a confounder, since carious dentine is inherently more permeable to dye than sound dentine and would have artificially inflated microleakage scores in the Hall and modified Hall groups relative to conventional preparation. Moosavi et al. found PMC internal adaptation to be unaffected by an underlying composite restoration [22], so the restoration step is unlikely to have biased the fit results.

The findings demonstrate that the preparation technique, rather than the luting cement, governed both the seal and the regional internal adaptation of the crown. The null hypotheses were therefore partially rejected—rejected for the effect of technique on microleakage and on the angular internal gap, but retained for the effect of cement and for the technique–cement interaction across all outcomes.

Marginal gap did not differ significantly among the three techniques. This is consistent with the prefabricated nature of PMCs, whose form can be adjusted only slightly by contouring and crimping, performed identically regardless of preparation technique, and whose standardised geometry relies on crimping and cement thickness more than on conformity to individual tooth anatomy for marginal sealing [22]. In cast metal crowns, the cervical finish-line configuration is itself a crucial determinant of marginal adaptation, the marginal gap differing most when the finish-line design differs [23]. PMCs, however, present no differentiated buccal or lingual cervical finish line, regardless of the preparation technique, to drive such a difference; they differed instead in occlusal and proximal reduction.

By contrast, internal adaptation was region-dependent. The occlusal region showed the widest gap in all groups, whereas the axial region was narrowest. A comparable regional pattern—the occlusal region being least well adapted—has been reported for precision-fabricated zirconia crowns [18], suggesting the occlusal region is inherently difficult to adapt across crown systems, here through the fixed internal contour of a PMC rather than through fabrication constraints. Notably, occlusal gap did not differ by technique: occlusal reduction adjusts seating height, not the crown’s fixed internal contour, so a wide occlusal cement space persisted across all techniques. Technique effects were instead localised to the angular region, where the Hall technique produced a significantly larger gap than conventional preparation. Of the three techniques, only conventional preparation bevels the occluso-buccal and occluso-lingual line angles. Because PMCs are typically seated lingual-first, with pressure applied buccally so the crown slides over the buccal surface into the gingival sulcus [5], reducing these angular areas may ease the crown’s passage over the line angles and allow better fit at the axio-occlusal angles. Whether incorporating an occlusal bevel into the modified Hall technique would enhance adaptation warrants investigation in future studies.

Microleakage followed the same trend. The Hall technique produced significantly greater microleakage than conventional preparation, with the modified Hall technique being intermediate and not significantly different from either. This ordering reinforces that preparation technique is the dominant influence on the marginal seal. This finding agrees with Erdemci et al. [14] and Thakur et al. [15], who reported that Hall-placed crowns leaked more than conventionally placed ones. Consistent with this, teeth with insufficient Hall-crown fit have shown markedly lower success, with major failure in 40% of cases [9]. Poor marginal fit permits leakage, allowing a sealed carious lesion to reactivate and progress to failure.

In contrast to the technique effect, the luting cement had no significant effect on any outcome. Earlier work has compared the microleakage of a range of luting cements under PMCs—including zinc phosphate, zinc polycarboxylate, glass ionomer, RMGIC, and resin cements [4,14,16,24,25,26]—but bioactive and bioceramic cements have not previously been tested in this application, so direct comparison with prior findings is not possible for those two materials.

The three cements performed comparably, with ACTIVA^™^ and Calibra^®^ Bio matching the well-established RMGIC. Calibra^®^ Bio shares the calcium aluminate–glass ionomer chemistry of Ceramir^®^ C&B, under which name the manufacturer markets it in some regions [27], so the more extensively studied Ceramir^®^ provides the closest available evidence for this cement. The sealing mechanism proposed for these calcium aluminate cements is the surface precipitation of hydroxyapatite, formed as dissolved calcium aluminate reprecipitates with phosphate from saliva, pulpal fluid, or the tooth, thereby occluding the marginal cement gap; this apatite formation is phosphate-dependent [28]. In the present saliva-free model, any sealing contribution the bioceramic derives from that mechanism in vivo would therefore be under-expressed, so its comparable rather than superior performance is better read as the absence of a demonstrable advantage under these conditions than as evidence against a clinical sealing benefit.

The calcium aluminate and ceramic ionomer chemistry of these bioceramic cements also carries possible liabilities—a tendency to dissolve, low mechanical strength, and weak dentin bonding, similar to conventional glass ionomers—whereas ACTIVA^™^’s ionic resin matrix lowers solubility and forms a chemical bond with dentin. This dentin bond underlies its reported sealing advantage on dentin-bonded restorations. Farah found that in zirconia crowns ACTIVA^™^ produced significantly lower microleakage than the calcium aluminate bioceramics Ceramir^®^ and Calibra^®^ Bio, most clearly at the dentin deep margin [11], while Vohra et al. reported that in dentin-bonded ceramic crowns ACTIVA^™^ gave significantly lower microleakage than both glass ionomer and resin cements [29]. This advantage, however, is a dentin-surface phenomenon: ACTIVA^™^ is a polymeric bioactive agent of silica-glass particles in an ionic polymeric matrix carrying calcium, phosphate, and fluoride ions, and in the presence of moisture it exchanges these ions with the residual apatite of the dentin surface, replacing the hydroxyl groups of its phosphate-acid matrix with calcium from dentin to form a robust chemical bond and a tight marginal seal that reduces dentinal sensitivity and microleakage [30]. In the present study, by contrast, microleakage was assessed only on the buccal and lingual surfaces, which remained unprepared enamel across all three techniques; the sole reduction there—a minimal bevel at the occluso-buccal and occluso-lingual line angles—was confined to enamel and exposed no dentin. With no dentin at the measured interface, the dentin-dependent bonding mechanism that distinguishes ACTIVA^™^ under ceramic crowns could not be expressed, so it sealed no better than the RMGIC or the bioceramic cement.

The reduced microleakage reported for RMGIC is likewise attributed partly to resin-monomer penetration into demineralised dentin forming a micromechanical bond [31], a mechanism equally inapplicable to the intact enamel measured here; on enamel, therefore, all three cements fall back on the ionic interaction they share with the mineralised substrate, consistent with the cement equivalence observed. This substrate account complements the restoration itself: where a PMC is retained mechanically by crimping and seated against a metal intaglio rather than a bonded ceramic surface, the solubility and dentin-bond differences that separate these cements under ceramic crowns carry little weight, and the seal is governed instead by preparation. By extension, full-coverage restorations whose preparation extends into dentin—notably prefabricated zirconia crowns—would present the dentin substrate on which these cements’ bonding chemistry is expressed; whether the bioactive and bioceramic cements differ in marginal seal under such crowns warrants direct assessment.

No significant correlation was found between microleakage and marginal gap or any internal region. This indicates that marginal discrepancy and leakage are not always directly correlated, and it indicates that microleakage in PMCs is governed not by the size of the marginal gap alone but by the integrity of the cement seal along the entire crown–tooth interface. Consistent with this, cement-space thickness influences the seal in a non-monotonic manner—narrower spaces do not guarantee less leakage, and no consensus “ideal” dimension exists [32,33]—so a wider gap may not translate into proportionally greater microleakage.

Several limitations should be noted. As an in vitro study, it could not reproduce the intraoral environment, including salivary flow and bite forces, which may affect crown seating, and it potentially overestimates microleakage, since dye may penetrate spaces that oral fluids cannot [19,20]. Furthermore, dye penetration measures linear rather than volumetric leakage, which may not fully capture microleakage under clinical conditions. It also relies on visual assessment, introducing some subjectivity [34]. Also, the pH of the dye solution was not measured directly; the dye is mildly acidic (manufacturer-specified pH 3.0–4.5), and although all specimens were immersed simultaneously under identical conditions, an effect on the luting interface cannot be excluded. Thermocycling was limited to 500 cycles, which is consistent with published microleakage studies of PMCs [4,19,20] and simulates a short intraoral service period; more extensive cycling would impose a greater hydrolytic challenge on the cement interface, and the present findings therefore reflect early rather than long-term seal performance. Storage and thermocycling in distilled water rather than a phosphate-containing medium may have suppressed the hydroxyapatite-forming mechanism attributed to the bioceramic cement, as discussed above. Caries, where present, was removed in all specimens to standardise the substrate and assess the mechanical consequences of the three preparation techniques, whereas in clinical Hall and modified Hall practice carious dentine is sealed in place. The interface tested here is consequently more uniform than the clinical situation, and the external validity of the microleakage findings for crowns seated directly over carious dentine is correspondingly limited. Crowns were seated by finger pressure, which is not quantifiable and may have varied between specimens, and the subsequent static load of 40 g was lower than in comparable in vitro studies, as no loading jig was available. Absolute gap values may therefore exceed those achievable under standardised higher loads, although the identical protocol and single operator across all groups mean that relative comparisons are unaffected. First and second primary molars were pooled without stratification. Because first primary molars show greater variability in crown dimensions, especially their cervical buccal bulges, they may achieve a less consistent crown fit than second molars [9,10], a difference reported to manifest as more frequent fit-related failure under the modified Hall technique; tooth type may therefore have contributed unmeasured variability to the fit and microleakage results. Sectioning was confined to the buccolingual plane, consistent with established methodology for assessing fit and microleakage under PMCs [4,19]. This plane does not capture three-dimensional adaptation and excludes the mesial and distal surfaces; as proximal reduction is the defining feature of the modified Hall technique, its effect on fit and seal was not directly assessed, and the intermediate position of this group reflects performance at the buccal and lingual interfaces. Finally, although the sample size was adequate for the large main effect it was calculated to detect (f = 0.60), the nine technique–cement cells contain only five specimens each, limiting sensitivity to interaction effects and modest cement-related differences; the absence of a significant cement effect should therefore be interpreted as a failure to demonstrate a difference rather than as evidence of equivalence, as a Type II error cannot be excluded.

Clinically meaningful evidence will require randomised trials comparing the three techniques in children, stratified by molar type, with major failure (loss of restoration, pulpal pathology, or irretrievable caries progression) as the primary outcome over at least two to three years, rather than a surrogate measure. Testing a modified Hall approach incorporating a defined occluso-axial bevel would allow the angular-adaptation deficit identified here to be evaluated clinically, and three-dimensional assessment such as confocal laser scanning microscopy, or sectioning in both the buccolingual and mesiodistal planes, would capture crown adaptation more completely and allow the effect of proximal reduction to be quantified directly. Establishing whether these cements are genuinely interchangeable would require studies designed specifically to test equivalence.

## 5. Conclusions

Within the limits of this in vitro study, the following conclusions can be drawn:

1. The preparation technique, rather than cement type, was the principal determinant of marginal seal and angular internal fit under PMCs, with conventional preparation superior to the Hall technique in both; the modified Hall technique was intermediate.
2. With respect to marginal seal and fit, the bioactive cement (ACTIVA^™^) and the bioceramic cement (Calibra^®^ Bio) performed comparably to RMGIC under PMCs across all preparation techniques. Whether this translates into clinical equivalence requires evaluation under dynamic loading and intraoral conditions.
3. Clinical trials with patient-relevant failure outcomes are needed to validate these findings, including evaluation of a modified Hall technique incorporating a defined occluso-axial bevel.

## Figures and Tables

**Table 1:** Luting cements evaluated in the study.

Subgroup	Material (Brand)	Type/Classification	Principal Composition	Manufacturer
A	ACTIVA^™^ BioACTIVE-CEMENT	Bioactive ionic resin-matrix cement (dual-cure)	Fluoraluminosilicate glass, silanated nonreactive fillers, methacrylate monomers, polyacrylic acid, water, initiators (chemical and light) *	Pulpdent Corp., Watertown, MA, USA
B	Calibra^®^ Bio	Bioactive calcium aluminate bioceramic cement (self-adhesive, self-cure)	Powder: calcium aluminate; glass powder; inert strontium fluoride Liquid: water; polyacrylic acid; tartaric acid; lithium chloride; nitrilotriacetic acid/trisodium salt	Dentsply Sirona, Charlotte, NC, USA
C	Riva Luting Plus	Resin-modified glass ionomer cement (self-cure)	Glass ionomer with proprietary ionglass reactive glass filler; high fluoride release and recharge; chemically bonds to tooth structure and metal; BPA/HEMA-free *	SDI Limited, Bayswater, Victoria, Australia

* From Huang et al. [17] and manufacturers; BPA: Bisphenol A, HEMA: 2-hydroxyethyl methacrylate.

**Table 2:** Descriptive statistics (mean ± SD) for marginal and internal (axial, angular, occlusal) gap and microleakage by preparation technique and luting cement *.

	Preparation Technique			Luting Cement		
Outcome	Conventional	Hall	Modified Hall	RIVA Luting Plus	Activa^™^	Calibra^®^ Bio
Marginal gap (mm)	0.38 ± 0.19	0.33 ± 0.16	0.31 ± 0.13	0.37 ± 0.18	0.31 ± 0.19	0.34 ± 0.12
Axial gap (mm)	0.44 ± 0.15	0.53 ± 0.18	0.47 ± 0.09	0.55 ± 0.18	0.46 ± 0.13	0.44 ± 0.11
Angular gap (mm)	0.39 ± 0.12 ^a^	0.66 ± 0.27 ^b^	0.52 ± 0.19 ^a,b^	0.58 ± 0.28	0.49 ± 0.18	0.50 ± 0.22
Occlusal gap (mm)	0.58 ± 0.21	0.61 ± 0.36	0.57 ± 0.20	0.58 ± 0.25	0.62 ± 0.27	0.57 ± 0.27
Microleakage (%)	13.37 ± 5.36 ^a^	23.95 ± 14.73 ^b^	18.65 ± 9.88 ^a,b^	18.82 ± 7.37	20.41 ± 13.41	16.75 ± 12.73

* Within a row, values sharing a superscript letter did not differ significantly. Angular gap: Hall > conventional, *p* = 0.004; microleakage: Hall > conventional, *p* = 0.045. Superscripts are shown only where the omnibus test was significant; no significant differences were found among luting cements for any outcome.

## Data Availability

The original contributions presented in this study are included in the article/Supplementary Material. Further inquiries can be directed to the corresponding author.

## Supplementary Materials

The following supporting information can be downloaded at https://www.mdpi.com/article/10.3390/children13091187/s1; CRIS Guidelines (Checklist for Reporting In-vitro Studies) [35,36,37].

## Abbreviations

The following abbreviations are used in this manuscript:

PMCs	Preformed metal crowns
AAPD	American Academy of Pediatric Dentistry
RMGIC	Resin-modified glass ionomer cement
BPA	Bisphenol A
HEMA	2-hydroxyethyl methacrylate
