# Supplementary material for: Marginal Fit, Internal Adaptation, and Microleakage of Preformed Metal Crowns: Impact of Three Preparation Techniques and Three Ion-Releasing Cements
Source: Children (Basel). 2026 Sep 3;13(9):1187. doi: 10.3390/children13091187 (PMC13605802; doi:10.3390/children13091187)
Supplement: Supplementary file 1 [file children-13-01187-s001.zip › children-4475390-supplementary.pdf]

CRIS Guidelines (Checklist for Reporting In-vitro Studies)

Section/Topic	Item No	Checklist item	Reported on page No
**Title and abstract**			
	1a	Identification as an in vitro/laboratory study in the title	1
	1b	Structured summary of trial design, methods, results, and conclusions	1–2
**Introduction**			
Background and objectives	2a	Scientific background and explanation of rationale	2–3
	2b	Specific objectives or hypotheses	3
**Methods**			
Interventions	3	The intervention for each group, including how and when they were actually administered, with sufficient detail to allow replication	3-8
Outcomes	4	Completely defined pre-specified primary and secondary outcome measures, including how and when they were assessed	3-8
Sample size	5	How sample size was determined	3
Randomisation: Sequence generation	6	Method used to generate the random allocation sequence	4
Allocation concealment mechanism	7	Mechanism used to implement the random allocation sequence (such as sequentially numbered containers), describing any steps taken to conceal the sequence until interventions were assigned	NA
Implementation	8	Who generated the random allocation sequence, who enrolled teeth, and who assigned teeth to intervention	NA
Blinding	9	If done, who was blinded after assignment to interventions (for example, care providers, those assessing outcomes) and how	6
Statistical methods	10	Statistical methods used to compare groups for primary and secondary outcomes	7-8
**Results**			
Numbers analysed	11a	For each group, number of 'items' included in each analysis and whether the analysis was by original assigned groups	8,9 (Table 2)
Outcomes and estimation	11b	For each primary and secondary outcome, results for each group, and the estimated effect size and its precision (such as 95% confidence interval)	8,9 (Table 2), (Figures 3–4)
**Discussion**			
Limitations	12a	Trial limitations, addressing sources of potential bias, imprecision, and, if relevant, multiplicity of analyses	10-13
Generalisability	12b	Generalisability (external validity, applicability) of the trial findings	10-13
Interpretation	12c	Interpretation consistent with results, balancing benefits and harms, and considering other relevant evidence	10-13
**Other information**			
Protocol	24	Where the full trial protocol can be accessed, if available	NA
Funding	25	Sources of funding and other support (such as supply of drugs), role of funders	14

Sources

[35] Krithikadatta, J.; Gopikrishna, V.; Datta, M. CRIS Guidelines (Checklist for Reporting In-vitro Studies). *J. Conserv. Dent.* **2014**, *17*, 301–304.
[36]. Faggion, C.M., Jr. Guidelines for reporting pre-clinical in vitro studies on dental materials. *J. Evid. Based Dent. Pract.* **2012**, *12*, 182–189.
[37] Available online: www.consort-statement.org (accessed on 15 July 2026)
